# A Pre- and Within-Pandemic Survey of SARS-CoV-2 RNA in Saliva Swabs from Stray Cats in Switzerland

**DOI:** 10.3390/v14040681

**Published:** 2022-03-25

**Authors:** Evelyn Kuhlmeier, Tatjana Chan, Julia Klaus, Benita Pineroli, Esther Geisser, Regina Hofmann-Lehmann, Marina L. Meli

**Affiliations:** 1 Clinical Laboratory, Department of Clinical Diagnostics and Services, and Center for Clinical Studies, Vetsuisse Faculty, University of Zurich, Winterthurerstrasse 260, 8057 Zurich, Switzerland; tchan@vetclinics.uzh.ch (T.C.); jklaus@vetclinics.uzh.ch (J.K.); bpineroli@vetclinics.uzh.ch (B.P.); rhofmann@vetclinics.uzh.ch (R.H.-L.); mmeli@vetclinics.uzh.ch (M.L.M.); 2 NetAP—Network for Animal Protection, Vogelsangstrasse 32, 8133 Esslingen, Switzerland; info@netap.ch

**Keywords:** SARS-CoV-2, RT-qPCR, stray cats, Switzerland, COVID-19, One Health, animal welfare

## Abstract

Cats have been shown to be highly susceptible to SARS-CoV-2, and transmission within the species has been demonstrated experimentally. In cats undergoing natural SARS-CoV-2 infections, human-to-animal transmission was mostly suspected. It can be postulated that, in stray cats with no or only minimal contact with humans, SARS-CoV-2 may pose a minor risk. The current study investigated the prevalence of active SARS-CoV-2 infections in Swiss stray cats using quantitative reverse transcriptase real-time polymerase chain reaction (RT-qPCR). Saliva swabs from 1405 stray cats were collected in 14 Swiss cantons. The animals were sampled between February 2019 and February 2020 (pre-COVID-19 cohort: 523 cats) and between February 2020 and August 2021 (COVID-19 cohort: 882 cats). All the samples were tested by RT-qPCR, amplifying the envelope (E) gene and, in case of positive or inconclusive results, the RNA-dependent RNA polymerase (RdRp) gene of SARS-CoV-2. No SARS-CoV-2 viral RNA could be detected in any of the tested saliva swab samples. Our findings support the assumption that SARS-CoV-2 infections in stray cats are not highly prevalent in Switzerland. Nevertheless, the monitoring of stray cats and other susceptible animal species is necessary, since the “One Health” approach has been recognized as being essential to successfully fight the COVID-19 pandemic.

## 1. Introduction

SARS-CoV-2 has spread throughout the globe since its first appearance in China in December 2019. In humans, 279,114,972 confirmed cases of infection worldwide and 5,397,580 cases of death have been reported (by the specified date of 27 December 2021) [1].

The origin of SARS-CoV-2 remains unclear, but, from all the different theories that have been hypothesized, animals are supposed to play a key role [2]. The infection of humans is most likely a result of one or more spillover events; therefore, continuous surveillance of the animal kingdom is of primary importance in a “One Health” context.

The high susceptibility of cats to SARS-CoV-2 has been proven in several experimental studies [3,4,5]. In one of these studies, the viral shedding in nasal swabs reached a peak on day 3 post-infection in the inoculated cats, and on day 7 post-exposure in the saliva of contact cats [4]. The high susceptibility is probably due to the ACE2 receptor in cats, which contains suitable contacting residues for the efficient binding of SARS-CoV-2 [6]. Airborne transmission [3] and transmission through direct contact between cats has been documented in experimental studies [4,7].

Early in the human pandemic, natural infections of cats were documented [8,9,10,11] and, since then, 102 cases have been reported worldwide to the World Organization for Animal Health (OIE) (Situation Report 5) [12].

In contrast to different survey studies, where SARS-CoV-2 RNA could be detected in 0.26% (1/387) [13] and 0.38% (1/260) [14] of cats, regardless of if they belonged to COVID-19-affected households, a larger proportion of cats up to 17.6% (3/17) were found to be SARS-CoV-2-positive by RT-qPCR when their owners had a confirmed history of SARS-CoV-2 infection [15]. Therefore, in agreement with the sequencing results of viruses from cats and SARS-CoV-2 genomes from humans of the same region, a human-to-feline transmission is the most likely route of infection in owned domestic cats [11,15]. According to a recent study, the infection risk for pets living in COVID-19-confirmed households is up to eight times higher than for pets without exposure [16].

Stray cats in Switzerland have almost no contact with people; nevertheless, they often live in close proximity to humans; they are sometimes fed by them, search through human food waste, or may come into contact with owned cats. Moreover, although stray cats can live solitarily, large numbers of them gather around areas where food is provided; therefore, these cats are regularly in close contact with other cats [17]. In contrast to owned cats, stray cats are at a higher risk of becoming infected and maintaining infections within their populations with pathogens, such as feline immunodeficiency virus (FIV) or feline leukemia virus (FeLV) [18]. This is due, particularly, to a lack of preventative care. For these reasons, stray cat populations may become an infection reservoir [19]. Especially in fertile cats, such as the cats in the current study at the time of sampling, an increased agonistic behavior was observed, which was associated with increased aggressive behavior against other cats, thus relating to a higher risk of infection transmission [20].

SARS-CoV-2 has been documented in stray animals; three feral cats (3%) as well as one dog (8%) tested RT-qPCR-positive in a study in the Netherlands [21]. Additionally, eleven cats (18%) and two dogs (15%) of the tested animals in this study had SARS-CoV-2 antibodies by virus neutralization assay. However, these animals lived under special conditions, around infected mink farms in the Netherlands. The authors assumed a mink-to-cat transmission of SARS-CoV-2 as the most likely scenario in these cases, which constituted one of the first natural animal interspecies transmissions [21]. In Rio de Janeiro, Brazil, two stray animals, a cat and a dog, captured during an effort to neuter and medically support abandoned animals, tested positive for SARS-CoV-2 antibodies in a plaque-reduction neutralization test, whereas the rectal and oropharyngeal swabs of all tested 96 animals were RT-qPCR-negative [22]. In a study in northern Italy, 99 stray cats were sampled; blood samples as well as interdigital, cutaneous, oropharyngeal, nasal, and rectal swabs were collected and tested negative in RT-qPCR and in serological examinations [23]. Another Italian study examined stray cats in Lombardy. As a result, one of 105 cats tested antibody-positive against SARS-CoV-2, but no cat showed an active infection [24]. Thus, so far, only limited exposure of stray cats to SARS-CoV-2 has been observed; however, only few studies have been conducted and the number of animals per study was limited.

The aim of this study was to assess active SARS-CoV-2 infection reflected by the shedding of viral RNA in a large population of Swiss stray cats. For this purpose, saliva swabs collected between 2019 and 2021 from 1405 cats were analyzed using RT-qPCR. The presence of active infection in these cats and the spread of infection within the population could indicate an important role of stray cats during the COVID-19 pandemic.

## 2. Materials and Methods

### 2.1. Study Setup and Sample Collection

In total, 1405 saliva samples from Swiss stray cats were collected. Sample collection was conducted for routine diagnostic purposes in the scope of neutering campaigns of the Swiss Network for Animal Protection (NetAP). Veterinarians were instructed how to collect the samples to avoid possible contamination and how to label them to allow a correct identification. Samples were collected from 17 February 2019 to 6 August 2021. The date of 24 February 2020 was chosen as a cut-off date between the pre-COVID-19 and the COVID-19 cohorts, the day before the first human COVID-19 case in Switzerland was documented [25].

Where available, data on the age, gender and health status of the cats were compiled. The age of the animals was estimated by the attending veterinarians, and a classification of age group was performed as follows: cats ≤ one year (junior); cats from > one year to <10 years (adult); and cats ≥ ten years (senior).

**Figure 1:**
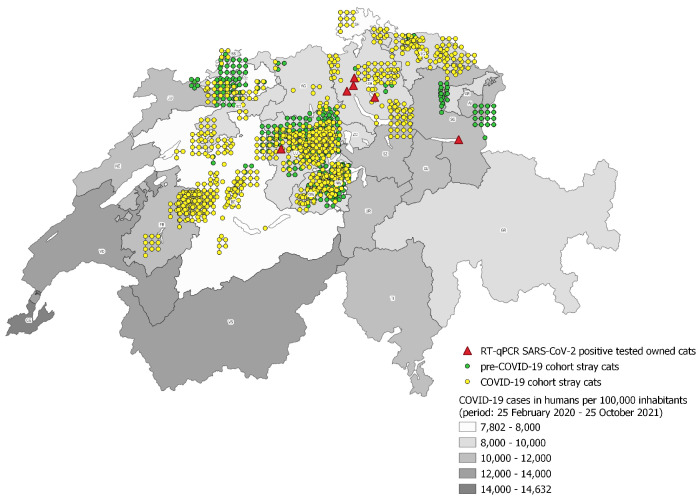
Geographic distribution of sampled stray cats of the COVID-19 cohort (yellow dots) and the pre-COVID-19 cohort (green dots). Coordinates for 39 samples are not reported (not indicated in the Figure; this includes 16 cats with unknown canton and coordinates and 23 with known canton but unknown coordinates). The RT-qPCR SARS-CoV-2-positive owned Swiss cats reported to the OIE-WAHIS are marked as red triangles [26]. The background colors (grey scale) reflect the infection rates of humans per 100,000 inhabitants (period from 25 February 2020 to 25 October 2021) in the individual cantons.

The health status of the cats was classified as healthy or sick according to the assessment of the attending veterinarians. Moreover, the attending veterinarians registered when a female cat was pregnant. Age, sex, health, and pregnancy status are given for all cats, for both the pre-COVID-19 and the COVID-19 cohorts, in Table 1. The samples originated from cats from the following 14 Swiss cantons: Lucerne (376 samples), Bern (178 samples), Nidwalden (158 samples), Thurgau (133 samples), Obwalden (115 samples), Solothurn (107 samples), Zurich (73 samples), Fribourg (76 samples), Aargau (71 samples), St. Gallen (61 samples), Basel-Country (22 samples), Schaffhausen (10 samples), Basel-City (8 samples), and Appenzell Outer-Rhodes (one sample); a total of 16 samples were of unknown origin (Figure 1).

Cotton swabs (Milian, Boswil, Switzerland; Brand, Divers/Dutscher; part no.: 020310) were used to collect saliva samples from all cats. Most of the cats were sampled under anesthesia during neutering, and sampling was conducted by veterinarians or veterinary technicians according to a provided protocol. The swabs were transferred to 1.5 mL tubes (Eppendorf AG, Hamburg, Germany) and the tubes were shipped at ambient temperature to the Clinical Laboratory, Vetsuisse Faculty, Zurich, for the analyses.

### 2.2. Sample Processing

Upon arrival, 200 µL of HBSS (Hanks’ balanced salt solution, without CaCl_2_ and MgCl_2_; GIBCO, Paisley, UK) was added to each tube. The samples were subjected to heat treatment (42 °C) for 10 min on a shaking incubator at 600 rpm, followed by centrifugation at 8000 rpm for one minute. To remove all the material from the swab, the swab was inverted in the tube using tweezers sterilized before and between every step with RNAse AWAY™ (Thermo Fisher Scientific, Basel, Switzerland) and ethanol (70%). With the cotton side towards the lid, the swab was centrifugated again for one minute at 8000 rpm, as previously described [27]. Afterwards, the swab was removed from the tube with the aid of sterile tweezers and the liquid part was put into a screw lid tube. All steps were performed in a laminar flow cabinet.

Before total nucleic acid (TNA) extraction, the samples were pooled with eight (8 × 25 µL) or 12 (12 × 16.66 µL) samples per pool, as described in detail by Studer et al. [28]. TNA extraction was carried out using a MagNA Pure LC 2.0 instrument with a MagNA Pure LC Total Nucleic Acid High Performance Kit (Roche Diagnostics AG, Rotkreuz, Switzerland), according to the manufacturer’s instructions. To monitor cross contaminations, a negative control (phosphate-buffered saline (PBS), without Ca^2+^ and Mg^2+^; Life Technologies Ltd., Paisley, UK) was included in every batch of extraction. The TNAs and the negative extraction control were stored at −20 °C until further processing.

The TNA samples were tested for the presence of SARS-CoV-2 RNA by RT-qPCR, as described previously [11,29]. All samples were first screened with the RT-qPCR targeting the SARS-CoV-2 envelope gene (E-gene). The samples with positive or inconclusive results were then additionally tested in the RNA-dependent RNA polymerase (RdRp) RT-qPCR assay. All the TNA samples were tested neat, as well as 1:5 diluted with RNAse–DNase-free water (AppliChem, Darmstadt, Germany) to detect possible inhibitions. A positive RT-qPCR control (RNA Wuhan RdRp-E-N; provided by the Swiss Federal Institute for Virology and Immunology, Mittelhäusern, Switzerland (IVI)), a negative control in the form of RNAse-DNase-free water, and a negative extraction control (PBS) were included for each RT-qPCR analysis [11].

RT-qPCR was considered positive if the cycle threshold (Ct) value was ≤38, questionable if the Ct value was >38 and ≤45, and negative with a Ct value > 45. A sample was judged as positive if a positive result occurred in both assays, E and RdRp RT-qPCR, as described in Klaus et al. [14].

### 2.3. Statistics

Pearson’s chi-squared test was performed to examine the differences in age between the cohorts (*p_Pearson_*). Fisher’s exact test was used to compare the frequency of sick animals and of animals with respiratory signs within the two cohorts (*p_Fisher_*). *p*-values < 0.05 were considered as significant.

## 3. Results

### 3.1. Sample Characteristics

Sample characteristics and geographic origin of all cats (1405) in the pre-COVID-19 cohort (523 cats) and the COVID-19 cohort (882 cats) are given in Table 1 and Figure 1.

In the COVID-19-cohort, there were significantly more adult cats (494/882; 56.0%) than in the pre-COVID-19 cohort (215/523; 41.1%; *p_Pearson_* < 0.00001). For 49 cats, the age was not registered (unknown).

Cats in the COVID-19 cohort were less frequently reported sick (15.1%) than cats in the pre-COVID-19 cohort (19.3%; *p_Fisher_* = 0.0454; Table 1). However, in the COVID-19 cohort, more cats with respiratory signs were reported (56/882; 6.3%) than in the pre-COVID-19 cohort (14/523; 2.7%; *p_Fisher_* = 0.0021; Table 2). For 73 cats, the health status was not registered (unknown).

### 3.2. SARS-CoV-2 Infection

In total, 21 saliva swab samples (10 cats from the COVID-19 cohort; 11 from the pre-COVID-19 cohort) tested positive in the E-gene RT-qPCR, but none of the 1405 saliva samples tested positive in both assays (the E-gene and the RdRp-gene RT-qPCR). Thus, none of the 1405 cats (0/523 of the pre-COVID-19 cohort; 0.00%; 95% CI: 0.00–0.70% and 0/882 of the COVID-19 cohort; 0.00%; 95% CI: 0.00–0.40%) were judged to be SARS-CoV-2-positive (i.e., both assays positive), as previously described [11].

## 4. Discussion

In the current study, the occurrence of active SARS-CoV-2 infection was investigated in 1405 stray cats, which were sampled during neutering campaigns in Switzerland from February 2019 to August 2021. The aim of the study was to assess the spread of active SARS-CoV-2 infections within the stray cat population in Switzerland. All 1405 feline saliva samples tested negative for SARS-CoV-2 viral RNA.

The same samples were also tested for FeLV viral RNA using a virus-specific RT-qPCR [30] (data not shown). FeLV is a retrovirus of domestic cats and closely related small wildcats (e.g., European wildcat, Iberian lynx) [31]; however, in contrast to SARS-CoV-2, it does not infect humans. Several of the 1405 samples tested positive for FeLV viral RNA (Hofmann-Lehmann et al. in preparation). This indicates that, although FeLV and SARS-CoV-2 are biologically different and show distinct pathogenesis, sample collection, shipping, processing and analyses were appropriate for the detection of viral RNA from viruses shed in the saliva of infected cats.

Since no SARS-CoV-2 RT-qPCR-positive cats were recognized, no conclusion as to whether the infection risk for SARS-CoV-2 in stray cats were influenced by sex, age, or comorbidities could be drawn. The cats in the COVID-19-cohort were more frequently adult cats; they were less frequently sick, but, if sick, they had respiratory signs more frequently; however, respiratory symptomatic cats also turned out to be SARS-CoV-2 negative, so no higher SARS-CoV-2 incidence was detectable in animals with respiratory signs.

All samples were taken by trained veterinarians or veterinary technicians and most of the cats were under anesthesia during the sampling procedure, so the expected error ratio of sampling was very low. The sample collection and assessment of the health status and age was carried out by different people, so deviations were possible and the estimated data varied depending on the performing veterinarian.

A limitation of the study may be that the samples were not collected in all cantons and not evenly distributed all over Switzerland. It may be argued that in some areas of Switzerland which were not sampled in this study, such as the cantons Valais, Vaud and Geneva, a higher incidence of human SARS-CoV-2 infections were present, as shown in Figure 1. This may theoretically reduce the chance of detecting SARS-CoV-2-positive stray cats within this study, and cantons with a high incidence of human infections should be included in future, especially because the main infection risk for domestic cats identified so far, according to sequencing analyses, comes from SARS-CoV-2-positive humans [11,15]. An Italian study provided evidence that the number of positive-tested pets correlates positively with infection in humans [32]. However, our study covered a large area of Switzerland and a large number of stray cats; therefore, it provides a good first overview. Further sample collection is currently ongoing.

It should be considered that the risk of infection for stray cats is not exclusively given by direct contact with humans, but also the human environment. Thereby, stray cats, which often live in the immediate surroundings of people, search for food in the waste, or drink contaminated water, cannot be excluded. Wastewater has tested SARS-CoV-2-positive by RT-qPCR before [33], and could potentially be a carrier, because even infection dynamics can be identified by the investigation of wastewater [34]; however, there is no evidence yet that contaminated wastewater could represent a source of infection.

Another possible transmission route, cat-to-cat transmission, has been experimentally proven [3]. Therefore, there could be a potential risk for stray cats to become infected by contact with owned domestic cats. Cat-to-cat transmission between owned and stray cats could not be proven in our study, although, by RT-qPCR, SARS-CoV-2-positive-tested owned cats were detected in the same cantons where stray cats were investigated, namely, Zurich (four positive owned cats), Lucerne (one positive owned cat) and St Gallen (one positive owned cat). These six cats were recorded as part of an ongoing study and reported to the World Organization for Animal Health (OIE-WAHIS) [26] by the Federal Food Safety and Veterinary Office, Switzerland (FSVO). One cat had proven outdoor access, one had no outdoor access and, for four of the SARS-CoV-2 infected owned cats, it was unknown whether they had access to the outdoors.

Although this study could not identify any active SARS-CoV-2 infection in Swiss stray cats, the virus has been found in stray animals before [21,22,24]. In some serological examinations, the number of positive-tested animals was higher (2/14; 14%) than in testing the same animals via RT-qPCR (0/14; 0%) [22]. Therefore, to complete the picture and obtain more detailed information, further studies should be conducted which also investigate the prevalence of antibodies against SARS-CoV-2 in the blood of stray cats. There is evidence that antibodies against SARS-CoV-2 could be detected for as long as 10 months, or even longer [35], in the blood of cats after infection. By RT-qPCR analyses only active infections are detected, and the longest duration of viral shedding during SARS-CoV-2 infection in cats has been identified as 21 days [36]. To generate data over an extended period, serological tests should be carried out. However, it needs to be noted that some serological studies in stray cats did not reveal any SARS-CoV-2-positive animals [23]. 

The investigation in active infections by RT-qPCR, as in the current study, is of importance in order to understand possible transmission routes and the duration of viral shedding. In the case of positive samples, this enables viral genome sequencing to identify new variants. Furthermore, despite the negative study results, the current study is of importance especially because of the large number of cats sampled, in view of the ubiquitous viral spread in the human population and the reporting of SARS-CoV-2 RT-qPCR-positive owned cats in Switzerland (Figure 1). Since none of the stray cats showed an active SARS-CoV-2 infection, the Swiss stray cat population seems to have a low risk to form a reservoir for SARS-CoV-2; therefore, the risk of development of new virus variants within this population, which relies on high virus replication rates, seems limited. This scientific knowledge of the distribution of SARS-CoV-2 in the animal kingdom is significant in consideration of “One Health”, and necessary to guarantee the safety of humans and animals.

## 5. Conclusions

In conclusion, none of the 1405 sampled stray cats tested SARS-CoV-2-positive by RT-qPCR. Our findings suggest that the spread of active SARS-CoV-2 infection in stray cat populations is limited. Nevertheless, further monitoring and serological investigations need to be performed to determine whether stray cats act as a virus reservoir.

## Figures and Tables

**Table 1:** Sample characteristics of all cats and cats categorized into the pre-COVID-19 and COVID-19 cohorts: number of animals, sex, age, health status, and presence of pregnancy.

		All Cats	Pre-COVID-19 Cohort	COVID-19 Cohort
Sampling period			From 17 February 2019 to 24 February 2020	From 25 February 2020 to 6 August 2021
Number of animals (*n*)		1405	523/1405 (37.2%)	882/1405 (62.8%)
Sex	Female	800/1405 (56.9%)	314/523 (60.0%)	486/882 (55.1%)
	Male	593/1405 (42.2%)	205/523 (39.2%)	388/882 (44.0%)
	Unknown	12/1405 (0.9%)	4/523 (0.8%)	8/882 (0.9%)
Age	Junior ^1^	627/1405 (44.6%)	272/523 (52.0%)	355/882 (40.2%)
	Adult ^2^	709/1405 (50.5%)	215/523 (41.1%) *	494/882 (56.0%) *
	Senior ^3^	20/1405 (1.4%)	8/523 (1.5%)	12/882 (1.4%)
	Unknown	49/1405 (3.5%)	28/523 (5.4%)	21/882 (2.4%)
Health status	Healthy	1098/1405 (78.1%)	364/523 (69.6%)	734/882 (83.2%)
	Sick	234/1405 (16.7%)	101/523 (19.3%) *	133/882 (15.1%) *
	Unknown	73/1405 (5.2%)	58/523 (11.1%)	15/882 (1.7%)
Pregnancy ^4^		28/800 (3.5%)	14/314 (4.5%)	14/486 (2.9%)

^1^ ≤one year; ^2^ from >one year to <10 years; ^3^ ≥10 years; ^4^ related to the number of female cats; * significant differences (Pearson’s chi-squared test/Fisher’s exact test, *p* < 0.05) between the cohorts are highlighted in bold font.

**Table 2:** Reported clinical signs in the sick-rated cats of both cohorts from February 2019 to August 2021.

Character of the Disease		All Cats^1,2^	Pre-COVID-19 Cohort ^1^	COVID-19 Cohort ^2^
Inflammatory	Respiratory	70/1405 (5.0%)	14/523 (2.7%) *	56/882 (6.3%) *
	Conjunctivitis	8/1405 (0.6%)	3/523 (0.6%)	5/882 (0.6%)
Traumatic	Injuries	17/1405 (1.2%)	13/523 (2.5%)	4/882 (0.5%)
Parasitic		48/1405 (3.4%)	33/523 (6.3%)	15/882 (1.7%)
Dental		52/1405 (3.7%)	22/523 (4.2%)	30/882 (3.4%)
Gastrointestinal	Diarrhea	19/1405 (1.4%)	7/523 (1.3%)	12/882 (1.4%)
Other/not reported		40/1405 (2.8%)	17/523 (3.3%)	23/882 (2.6%)

^1^ multiple answers for seven cats (six cats, two diseases; one cat, three diseases); ^2^ multiple answers for twelve cats (two diseases); * significant differences between the cohorts are highlighted in bold font (Fisher’s exact test, *p* < 0.05).

## Data Availability

All available data are presented in this manuscript.
